# Characterization of adrenal glands on computed tomography with a 3D V-Net-based model

**DOI:** 10.1186/s13244-025-01898-7

**Published:** 2025-01-14

**Authors:** Yuanchong Chen, Yaofeng Zhang, Xiaodong Zhang, Xiaoying Wang

**Affiliations:** 1 Department of Radiology, Peking University First Hospital, Beijing, 100034 China; 2 Beijing Smart Tree Medical Technology Co. Ltd., Beijing, 100011 China

**Keywords:** Adrenal gland, Computed tomography, Deep learning, Segmentation, Classification

## Abstract

**Objectives:**

To evaluate the performance of a 3D V-Net-based segmentation model of adrenal lesions in characterizing adrenal glands as normal or abnormal.

**Methods:**

A total of 1086 CT image series with focal adrenal lesions were retrospectively collected, annotated, and used for the training of the adrenal lesion segmentation model. The dice similarity coefficient (DSC) of the test set was used to evaluate the segmentation performance. The other cohort, consisting of 959 patients with pathologically confirmed adrenal lesions (external validation dataset 1), was included for validation of the classification performance of this model. Then, another consecutive cohort of patients with a history of malignancy (*N* = 479) was used for validation in the screening population (external validation dataset 2). Parameters of sensitivity, accuracy, etc., were used, and the performance of the model was compared to the radiology report in these validation scenes.

**Results:**

The DSC of the test set of the segmentation model was 0.900 (0.810–0.965) (median (interquartile range)). The model showed sensitivities and accuracies of 99.7%, 98.3% and 87.2%, 62.2% in external validation datasets 1 and 2, respectively. It showed no significant difference comparing to radiology reports in external validation datasets 1 and lesion-containing groups of external validation datasets 2 (*p* = 1.000 and *p* > 0.05, respectively).

**Conclusion:**

The 3D V-Net-based segmentation model of adrenal lesions can be used for the binary classification of adrenal glands.

**Critical relevance statement:**

A 3D V-Net-based segmentation model of adrenal lesions can be used for the detection of abnormalities of adrenal glands, with a high accuracy in the pre-surgical scene as well as a high sensitivity in the screening scene.

**Key Points:**

- Adrenal lesions may be prone to inter-observer variability in routine diagnostic workflow.
- The study developed a 3D V-Net-based segmentation model of adrenal lesions with DSC 0.900 in the test set.
- The model showed high sensitivity and accuracy of abnormalities detection in different scenes.

**Graphical Abstract:**

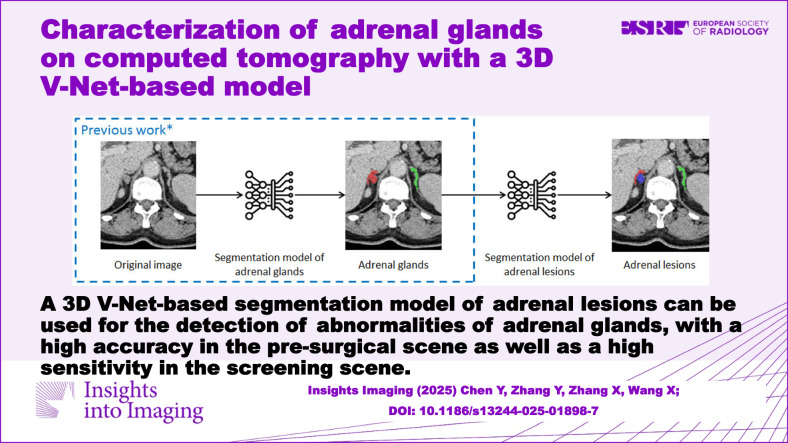

## Introduction

Adrenal disorders pose challenges in clinical diagnosis and management, necessitating accurate and efficient delineation of structural abnormalities. Computed tomography (CT) is the modality of choice for adrenal imaging [1, 2]. Focal adrenal lesions are often discovered incidentally. Incidentalomas are defined as adrenal masses with a diameter larger than 1 cm detected on cross-sectional imaging for an unrelated indication, and have a prevalence of 7% on CT [3, 4]. Traditional methods of adrenal gland analysis on CT images are prone to inter-observer variability and may lack precision in capturing subtle pathological changes [5].

In recent years, deep learning techniques have demonstrated remarkable success in tasks of medical imaging that have been applied to many organs in both CT and MRI [6–9], offering a promising avenue for automating and enhancing the accuracy of adrenal gland classification. Previous studies in differentiating normal and abnormal adrenal glands are often small-sampled, internally validated, or without pathological confirmation [10–13]. Studies with deep learning-based approaches and comprehensive external validation are needed to ensure the stability of the classification performance.

This study focuses on advancing the classification of adrenal glands using a 3D V-Net [14] segmentation approach for adrenal lesions and validates the model in pre-operational and screening cases. We set up a 3D V-Net-based segmentation model of adrenal lesions and evaluated its performance in characterizing adrenal glands as normal or abnormal.

## Materials and methods

### Patients

**Fig. 1:**
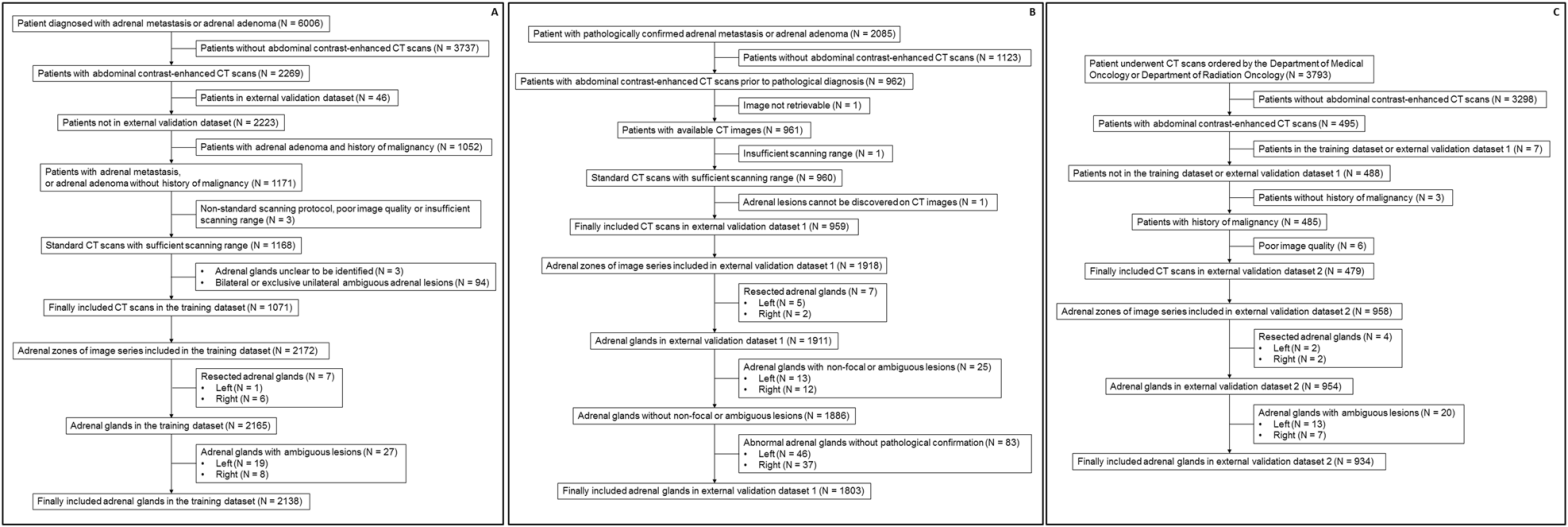
The inclusion and exclusion flowchart of patients and adrenal glands in the (**A**) training dataset, (**B**) external validation dataset 1, and (**C**) external validation dataset 2

This retrospective study was approved by the local institutional review board (No. 2023 (371)), with a waiver of informed consent. The inclusion and exclusion flowchart of the study cohort is shown in Fig. 1.

#### Training dataset

The contrast-enhanced abdominal CT (including portal venous phase (PVP) (images of patients diagnosed with focal adrenal lesions upon comprehensive clinical diagnosis were included after the retrieval of the Hospital Information System. The lesions in these patients were not confirmed by pathological diagnosis. The PVP was selected to train the segmentation model of adrenal lesions. These images were acquired by GE Discovery CT750HD, GE LightSpeed VCT, GE Optima CT680 Expert, GE Revolution CT, Siemens SOMATOM Definition Flash, Siemens SOMATOM Force, Philips Brilliance 64, Philips Brilliance iCT 256, and Neusoft NeuViz Prime.

The inclusion criteria were as follows: (1) patients diagnosed with “adrenal metastasis” from January 1st, 2012, to September 30th, 2022, or “adrenal adenoma” from January 1st, 2021, to September 15th, 2022, upon discharge and (2) patients who underwent contrast-enhanced abdominal CT scans in our center. The exclusion criteria were as follows: (1) patients with a history of malignancy who were diagnosed with “adrenal adenoma”; (2) images could not be retrieved; (3) poor image quality; (4) non-standard scanning protocol; (5) insufficient scanning range; (6) patients included in external validation dataset 1; (7) postoperative or abnormal changes that made the adrenal gland unclear to be identified; and (8) patients with bilateral or exclusive unilateral ambiguous lesions. The image series of multiple reconstructions from the same PVP scan were kept. All these images were reviewed by 2 radiologists, and adrenals with non-focal, ambiguous, or undiscovered lesions were excluded. Finally, 1086 image series with 2138 adrenals were enrolled for training (213 metastases and 1047 adenomas, Fig. 1A).

#### External validation dataset

Dataset 1: cases with pathologically confirmed focal adrenal lesions

Patients who underwent adrenalectomy or adrenal biopsy and were diagnosed with focal adrenal lesions pathologically were included, and the PVP image series of their most recent contrast-enhanced abdominal CT scans, as well as the corresponding radiology report before the procedure, were collected. These images were acquired by GE Discovery CT750HD, GE LightSpeed VCT, GE Optima CT680 Expert, GE Revolution CT, Siemens SOMATOM Definition Flash, Siemens SOMATOM Force, Philips Brilliance 64, Philips Brilliance iCT 256, and Neusoft NeuViz Prime.

The inclusion criteria were as follows: (1) patients diagnosed with focal adrenal lesions pathologically from January 1st, 2013, to October 11th, 2022, and (2) patients who underwent contrast-enhanced abdominal CT scans in our center. The exclusion criteria were those of the training set, plus: (1) multiple ipsilateral lesions with indistinguishable pathological results. Only one image series of the same patient was kept. Abnormal adrenal glands without pathological confirmation were excluded. After being reviewed by 2 radiologists, 959 image series (*N* = 944 from adrenalectomy, *N* = 15 from biopsy) with 1803 adrenals were enrolled for analysis (including 54 metastases and 647 adenomas, Fig. 1B).

Dataset 2: consecutive cases with a history of malignancy

The follow-up or baseline contrast-enhanced abdominal CT scans of patients who were diagnosed with malignancy that underwent or were in preparation for chemotherapy or radiotherapy were collected, and the PVP image series and the radiology report were consecutively included for analysis. These images were acquired by GE Discovery CT750HD, GE Optima CT680 Expert, GE Revolution CT, Siemens SOMATOM Definition Flash, Siemens SOMATOM Force, Philips Brilliance iCT 256, and Neusoft NeuViz Prime.

The inclusion criteria were as follows: (1) contrast-enhanced abdominal CT scans ordered by the Department of Medical Oncology or Department of Radiation Oncology and (2) exam date from May 1st, 2023, to June 30th, 2023. The exclusion criteria were those of the training set, plus: (1) patients without a history of malignancy according to the diagnosis recorded in the hospital information system and (2) patients included in the training dataset or the external validation dataset 1. After being reviewed by 2 radiologists, 479 image series with 934 adrenals were enrolled for analysis (Fig. 1C).

### Image acquisition

A contrast agent (iopromide 370 mgI/mL or iohexol 320 mgI/mL, Bayer AG) was injected through the venous system at a flow rate of 2.5 mL/s or 3 mL/s. The injection dose was 100 mL for patients weighing less than 75 kg, 120 mL for patients weighing 75–90 kg, and 150 mL for patients weighing more than 90 kg. All patients underwent a PVP scan (60–70 s after contrast injection). The tube voltage was 120 kVp, and auto-mAs were applied. Images were reconstructed with slice thickness of 1 mm or 1.25 mm and interval 1 mm.

### Segmentation of adrenal glands and adrenal lesions

**Fig. 2:**
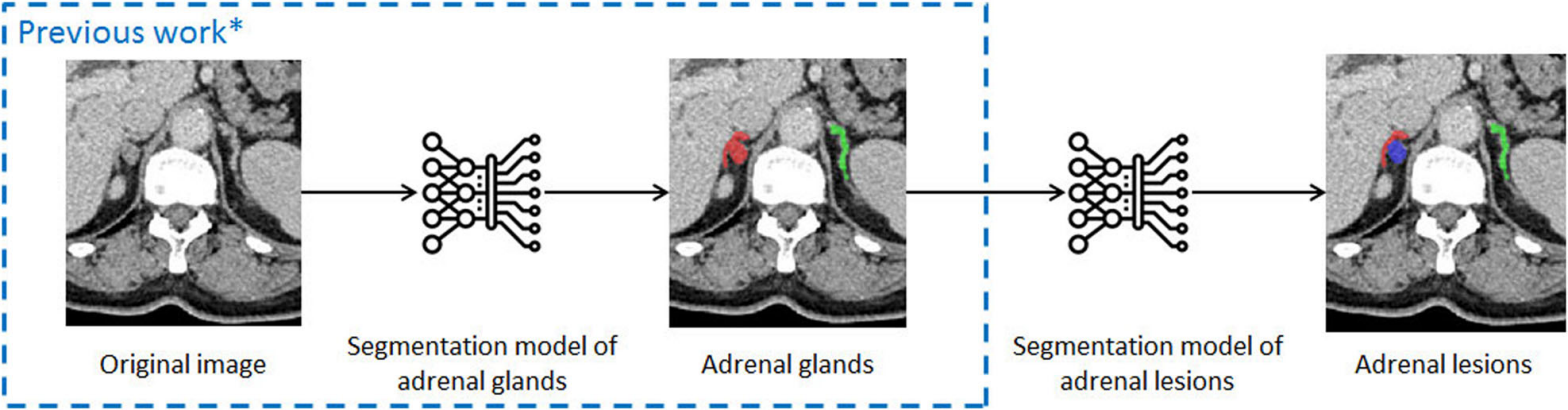
Overview of the workflow of the present study. CT images underwent segmentation of adrenal glands and subsequent segmentation of adrenal lesions. *The segmentation model of adrenal glands is previously developed [15]

The image series included in this study underwent autolabeling by the previously developed adrenal segmentation model [15] (Fig. 2). These bilateral adrenal labels were checked by a junior radiologist and a senior radiologist together, and unsatisfied labels were modified manually. Note that the adrenal labels of those after adrenalectomy were left empty. All CT images were reviewed by a junior radiologist and a senior radiologist together, and the normal/abnormal results were recorded after discussion. If no consensus could be reached, or they both could not determine whether any masses were on the adrenal gland, the result was given by a third senior radiologist (normal, abnormal, or ambiguous). If the third senior radiologist still did not have enough confidence to determine the lesion-containing status of the adrenal gland, it was marked as an “ambiguous gland” and excluded. The above procedure was used for all datasets in this study, including the training dataset and external validation datasets 1 and 2. In the training set or external validation dataset 1, patients with a unilateral ambiguous adrenal gland and another normal adrenal gland were excluded. Abnormal adrenal glands without pathological confirmation in external validation dataset 1 were not included for analysis. Then, focal adrenal lesions in these datasets were manually annotated within the adrenal label by one junior radiologist and checked by a senior radiologist if a focal lesion was discovered in the region of the adrenal gland.

### Model training and label prediction

In the training dataset, the image series were randomly allocated into training set (*N* = 870), validation set (*N* = 108), and test set (*N* = 108) to train the segmentation model of adrenal lesions.

This study was based on a 3D V-Net architecture [14] equipped with Nvidia Tesla P100 16 G (Nvidia Corporation, Santa Clara, CA) GPU and PyTorch v1.7.1 + cu110 (https://pytorch.org/). The model inputs segmented adrenal glands from PVP CT images and outputs focal adrenal lesions. The output of a normal adrenal gland will be an empty label. Image preprocessing included window adjustment (center 30 HU, width 300 HU), resizing to 128px × 192px × 256px, and image augmentation by rotating, sheering, noise injection, denoising, etc. The training parameters were batch size 6, learning rate 0.0001, and epoch 400.

The 3D V-Net-based segmentation model for adrenal lesions was used to predict adrenal gland abnormalities. Adrenals with segmented regions that have overlapping voxels with their actual lesions were considered abnormal. Image series were grouped according to the diameter of the containing largest lesion, i.e., the contralateral adrenal glands (if not excluded) were included in the corresponding group as well. Detection performance was analyzed in each group.

### Statistical analysis

Statistical analyses were performed using Python 3.7.6 with Scipy 1.4.1, Statsmodels 0.11.0, and Sklearn 0.22.1, unless otherwise specified. Dice similarity coefficient (DSC), Hausdorff distance (HD), and volumetric similarity (VS) were used to evaluate the segmentation performance. The reference standard was set as the annotation made by radiologists. Quantitative parameters of manually annotated adrenal gland and adrenal lesions were calculated programmatically. All analyses were two-sided, and the statistical significance was set at *p* < 0.05. Data normality was determined by the Kolmogorov‒Smirnov test. Continuous variables in univariate analysis were compared using Student’s *t*-test and the Mann–Whitney *U*-test, as appropriate. The reference standard for different classification methods (i.e., the model and the radiology report) was the result of image review by radiologists, and the McNemar test was used for comparisons between these classification methods.

## Results

### The segmentation model of adrenal lesions

Patient characteristics are shown in Table 1. A total of 1071 patients (2138 adrenal glands from 1086 image series) were used for model training, 164 of whom were diagnosed with adrenal metastasis. The incidence of abnormal adrenal glands was 58.9% (1260/2138). The most common malignancy was lung cancer (50/164, 30.5%) among those with adrenal metastasis, as shown in Table 1. The characteristics of adrenal lesions are shown in Table 2. The left and right-sided lesions were not significantly different except for the volumetric percentage of lesions in the adrenal gland (*p* = 0.020).

**Table 1:** Patient characteristics of datasets in this study

	Training dataset	External validation dataset 1	External validation dataset 2
Number of patients	1071	959	479
Males	647	486	272
Females	424	473	207
Age	61.0 (53.50–69.0)	51.0 (40.0–59.0)	61.0 (53.0–69.0)
Number of adrenal glands	2138	1803	934
Left	1066	895	464
Normal	325	384	379
Abnormal	741	511	85
Right	1072	908	470
Normal	553	451	430
Abnormal	519	457	40
Number of patients with history of malignancy	164	126	479
Breast	9	10	31
Colorectal	22	5	105
Esophageal	2	0	31
Gastric	12	0	36
HCC	13	4	0
Lung	50	18	40
RCC	23	62	24
TCC	5	8	46
Thyroid gland	0	11	0
Other	28	7	166
Unknown primary site	1	1	0

Data are described as median (interquartile range)

*HCC* hepatocellular carcinoma, *RCC* renal cell carcinoma, *TCC* transitional cell carcinoma

**Table 2:** Patient characteristics of external validation dataset 1

	Metastasis	Adenoma	Other types
Number of patients	53	646	260
Males	38	310	138
Females	15	336	122
Age	60.0 (53.0–67.0)	51.0 (42.0–59.0)	46.0 (34.0–56.0)
Number of adrenal glands	89	1211	503
Left	41	604	250
Normal	14	243	127
Abnormal	27	361	123
Right	48	607	253
Normal	21	321	109
Abnormal	27	286	144

Data are described as median (interquartile range)

The DSC, HD, and VS of the test set (*N* = 108) were 0.900 (0.810–0.965), 3.030 (1.805–8.115) mm, and 0.960 (0.860–0.990), respectively. Data were described as median (interquartile range).

### External validation of adrenal classification from the segmentation results

#### Cases with pathologically confirmed focal adrenal lesions

A total of 959 patients (1803 adrenals) were collected with pathologically confirmed adrenal metastasis (53/959, 5.5%), adenoma (646/959, 67.4%) or other pathological types (260/959, 27.1%; details of types are shown in Supplementary Table 1). Table 1 shows the malignancies in this group of patients. Note that 11.8% (76/646) of patients with adrenal adenoma had a history of malignancy. The maximum diameter, volume, and volumetric percentage of lesions in this dataset are significantly different from other datasets (all *p* < 0.001, Table 3).

**Table 3:** Characteristics of adrenal lesions in datasets

	Training dataset				External validation dataset 1				External validation dataset 2			
	All	Left	Right	*p*-value	All	Left	Right	*p*-value	All	Left	Right	*p*-value
Number of adrenal lesions^a^	1260	741	519	-	968	511	457	-	81	51	30	-
Maximum diameter/mm	17.79 (12.96–28.27)	17.82 (12.96–29.09)	17.78 (12.97–26.83)	0.202	32.87 (23.11–46.84)	32.23 (22.67–46.17)	33.46 (23.60–47.21)	0.122	17.88 (11.06–27.70)	17.49 (10.27–26.34)	19.46 (12.65–26.91)	0.696
Volume/mm^3^	926.91 (397.76–2755.89)	890.81 (399.43–2700.82)	1001.87 (397.36–2906.41)	0.234	7500.48 (2311.41–21,639.53)	7207.27 (2190.72–19,986.56)	7504.02 (2741.79–23,358.99)	0.154	879.23 (241.37–2128.35)	598.61 (221.00–1767.06)	1532.38 (340.46–3228.26)	0.106
Volumetric percentage of the adrenal gland (%)	22.77 (10.46–53.11)	21.99 (10.27–50.23)	24.46 (11.37–55.99)	0.020	82.59 (47.84–94.84)	81.31 (44.24–94.23)	83.26 (53.08–95.46)	0.055	23.30 (5.94–44.99)	18.49 (5.48–38.91)	36.29 (10.37–56.17)	0.052

Data are described as median (interquartile range)

^a^ For adrenal glands with multiple lesions, only the lesion with the largest volume was included for analysis in this table

The adrenal glands of these patients underwent prediction using the 3D V-Net-based segmentation model of adrenal lesions. Classification results of different groups of lesions are shown in Table 4. Note that the sensitivity and accuracy of the model are 98.3% and 99.7%, respectively, which showed no significant difference compared to radiology reports (*p* = 1.000), whose sensitivity and accuracy are 98.5% and 98.8%, respectively, of the whole dataset. There is no significant difference between the model and the radiology reports in each group, of the metastasis, adenoma, other pathological types, or the whole dataset (*p* = 1.000). The results of metastasis, adenoma, and other pathological types is shown in Supplementary Tables 2–4.

**Table 4:** Classification results of external validation dataset 1

Lesion range	Method	Total adrenal glands	Prevalence	Accuracy	Sensitivity	Specificity	PPV	NPV	*p*-value
All	Model	1803	0.537	0.983 (0.977–0.989)	0.997 (0.993–1.000)	0.968 (0.956–0.980)	0.973 (0.963–0.983)	0.996 (0.992–1.000)	1.000
	Report			0.985 (0.979–0.991)	0.998 (0.995–1.000)	0.970 (0.959–0.982)	0.975 (0.965–0.985)	0.998 (0.994–1.000)	
5–10 mm	Model	2	0.5	0.000 (0.000–0.000)	0.000 (0.000–0.000)	0.000 (0.000–0.000)	0.000 (0.000–0.000)	0.000 (0.000–0.000)	1.000
	Report			1.000 (1.000–1.000)	1.000 (1.000–1.000)	1.000 (1.000–1.000)	1.000 (1.000–1.000)	1.000 (1.000–1.000)	
10–15 mm	Model	81	0.519	0.963 (0.922–1.000)	0.976 (0.930–1.000)	0.949 (0.879–1.000)	0.953 (0.891–1.000)	0.974 (0.923–1.000)	1.000
	Report			1.000 (1.000–1.000)	1.000 (1.000–1.000)	1.000 (1.000–1.000)	1.000 (1.000–1.000)	1.000 (1.000–1.000)	
> 15 mm	Model	1720	0.538	0.985 (0.980–0.991)	0.999 (0.997–1.000)	0.970 (0.958–0.982)	0.975 (0.965–0.985)	0.999 (0.996–1.000)	1.000
	Report			0.984 (0.978–0.990)	0.998 (0.995–1.000)	0.969 (0.956–0.981)	0.974 (0.963–0.984)	0.997 (0.994–1.000)	

#### Consecutive cases with a history of malignancy

A consecutive cohort of patients with malignancy (*N* = 479) was used to validate the classification performance of the model. Colorectal cancer (105/479, 21.9%) was the most common among them. A total of 13.4% (125/934) of the included adrenals were abnormal. The volume (*p* = 0.038) and volumetric percentage (*p* = 0.045) of the lesions are significantly different from the training dataset except for the maximum diameter (*p* = 0.140), as shown in Table 3.

The detection performance of the model and the radiology report is shown in Table 5. In the whole dataset, the performance of the model differs from the radiology report (*p* < 0.001), but the model shows a considerable negative predictive value (NPV) of 96.7%. In groups of lesion-containing images, the model is not significantly different from the radiology report (*p* > 0.05). Of note, the model has the ability to detect diffused abnormalities of the adrenal gland that are not significantly different from the radiology report (*p* = 0.092), with sensitivity and accuracy of 77.3% and 81.5%, respectively.

**Table 5:** Classification results of external validation dataset 2

Lesion range	Method	Total adrenal glands	Prevalence	Accuracy	Sensitivity	Specificity	PPV	NPV	*p*-value
All	Model	927	0.135	0.622 (0.591–0.654)	0.872 (0.813–0.931)	0.584 (0.549–0.618)	0.246 (0.206–0.286)	0.967 (0.951–0.983)	< 0.001
	Report			0.977 (0.968–0.987)	0.920 (0.872–0.968)	0.986 (0.978–0.994)	0.913 (0.863–0.962)	0.988 (0.980–0.995)	
Normal (no lesion)	Model	731	0	0.551 (0.515–0.587)	-	0.551 (0.515–0.587)	0.000 (0.000–0.000)	1.000 (1.000–1.000)	< 0.001
	Report			0.989 (0.982–0.997)	-	0.989 (0.982–0.997)	0.000 (0.000–0.000)	1.000 (1.000–1.000)	
Diffused abnormality	Model	65	0.677	0.815 (0.721–0.910)	0.773 (0.649–0.897)	0.905 (0.779–1.000)	0.944 (0.870–1.000)	0.655 (0.482–0.828)	0.092
	Report			0.985 (0.955–1.000)	0.977 (0.933–1.000)	1.000 (1.000–1.000)	1.000 (1.000–1.000)	0.955 (0.868–1.000)	
0–5 mm	Model	1	1	0.000 (0.000–0.000)	0.000 (0.000–0.000)	-	-	0.000 (0.000–0.000)	1.000
	Report			1.000 (1.000–1.000)	1.000 (1.000–1.000)	-	1.000 (1.000–1.000)	-	
5–10 mm	Model	29	0.552	0.793 (0.646–0.941)	0.812 (0.621–1.000)	0.769 (0.540–0.998)	0.812 (0.621–1.000)	0.769 (0.540–0.998)	0.508
	Report			0.828 (0.690–0.965)	0.750 (0.538–0.962)	0.923 (0.778–1.000)	0.923 (0.778–1.000)	0.750 (0.538–0.962)	
10–15 mm	Model	27	0.593	0.963 (0.892–1.000)	0.938 (0.819–1.000)	1.000 (1.000–1.000)	1.000 (1.000–1.000)	0.917 (0.760–1.000)	1.000
	Report			0.963 (0.892–1.000)	0.938 (0.819–1.000)	1.000 (1.000–1.000)	1.000 (1.000–1.000)	0.917 (0.760–1.000)	
> 15 mm	Model	74	0.649	0.973 (0.936–1.000)	0.979 (0.939–1.000)	0.962 (0.888–1.000)	0.979 (0.939–1.000)	0.962 (0.888–1.000)	0.688
	Report			0.919 (0.857–0.981)	0.917 (0.838–0.995)	0.923 (0.821–1.000)	0.957 (0.898–1.000)	0.857 (0.728–0.987)	

## Discussion

Adrenal glands are tiny organs in the human body, and their abnormalities may be omitted in the routine diagnostic workflow. The present study included the most common benign and malignant lesions of the adrenal gland, adenoma, and metastasis [16] and developed a 3D V-Net-based segmentation model that can be used for the classification of normal and abnormal adrenal glands, with no significant difference compared to radiology reports. Omissions in imaging diagnosis are expected to be reduced with the help of the model, which may play a role in tumor staging as well as treatment planning for those lesions occurring in patients with malignancy [17].

Previous studies of adrenal classification have limited sample sizes. The random forest classification has been used for adrenal glands and achieved a high sensitivity and specificity of 80% and 90%, respectively, in a small dataset of 20 adrenal glands in total [10]. The U-Net-based segmentation model of adrenal glands and subsequent automatic measurement achieved good performance in differentiating adrenal hyperplasia from normal adrenal glands and was developed and validated using 308 CT examinations [11].

The main strength of this study is the external validation in two different clinical scenes. The sample size of pathologically confirmed adrenal lesions is also larger than that in previous similar studies. Prior to surgical or interventional procedures, the model showed a robust accuracy (98.3%) that was not significantly different from the radiology report. The model trained from clinically diagnosed metastasis/adenoma achieved a considerable performance in the pre-surgical scene, of pathologically confirmed metastasis/adenoma and other pathological types. However, this cohort of patients may not be a good representative of the screening population because of their potential ambiguity or causative symptoms that require removal or pathological confirmation. In the consecutive cohort with a history of malignancy whose incidence of abnormal adrenals is relatively low (13.4%, 125/934 in total), the performance of the model is quite different. The model showed lower sensitivity (87.2%) of the dataset. This result is similar to a previous study with a secondary test dataset with consecutive cases (*N* = 991) that showed a sensitivity of 69% [12].

In the screening scene, the model showed the ability to generalization from focal adrenal lesions to diffused lesions. The model is trained with only focal lesion-containing and lesion-free adrenal glands. When it comes to diffused lesions, the model shows the ability of such detection and the performance between the model and the radiology report is not significantly different (*p* = 0.092). Nevertheless, the model showed a high NPV among different groups, especially the whole dataset (96.7%), indicating the potential role of “rule-out” normal adrenal glands before reviewing the images that can help the radiologist focus on those potentially abnormal adrenals to some extent. Of note, the false positive rate of the group of normal adrenal glands (44.9%) is relatively high, which might be caused by the over-sensitive model that has a high prevalence of adrenal lesions in the training dataset. More normal adrenal glands are needed to train such models to reduce such false positives in further studies.

There are several limitations to this study. First, this single-center retrospective study may not be representative if different scanning protocols were used in other institutions. Second, ambiguous adrenal lesions were excluded from the analysis. These lesions may have the potential to change, which would become obvious afterward. Follow-up imaging of the same patient should be reviewed in further studies. Third, the training set needs to be enlarged, especially including more normal adrenal glands, to reduce the false positive cases in the screening scene, as mentioned above. The new model is also to be validated in the general population in the detection of adrenal abnormalities. Nevertheless, images of post-surgical adrenal glands should also be included for further application in the real-world setting. Fourth, the adrenal lesions in the training dataset and external validation dataset 2 were not confirmed pathologically due to limited cases with pathological results. Studies of fully confirmed abnormalities are also needed in the future.

In conclusion, a 3D V-Net-based segmentation model can be used for the classification of normal and abnormal adrenal glands that have considerable performance in both pre-operational and consecutive screening cases without previous adrenal surgery. It also showed the ability of generalization, from metastasis/adenoma to other pathological types, and from focal to diffused lesions.

## Supplementary information

ELECTRONIC SUPPLEMENTARY MATERIAL

## Data Availability

The datasets used and analyzed during the current study are available from the corresponding author upon reasonable request.

## Abbreviations

CT: Computed tomography
DSC: Dice similarity coefficient
HD: Hausdorff distance
NPV: Negative predictive value
PVP: Portal venous phase
VS: Volumetric similarity
