# Supplementary material for: Characterization of adrenal glands on computed tomography with a 3D V-Net-based model
Source: Insights Imaging. 2025 Jan 14;16:17. doi: 10.1186/s13244-025-01898-7 (PMC11732807; doi:10.1186/s13244-025-01898-7)
Supplement: Supplementary file 1 — ELECTRONIC SUPPLEMENTARY MATERIAL [file 13244_2025_1898_MOESM1_ESM.pdf]

Characterization of adrenal glands on computed tomography with a 3D V-Net-based model

ELECTRONIC SUPPLEMENTARY MATERIAL

Supplementary Table 1: Pathological types of adrenal lesions in external validation dataset 1

	**Number**
**Metastasis**	54
**Adenoma**	647
**Adrenocortical carcinoma**	11
**Pheochromocytoma**	131
**Cyst**	46
**Gangliocytoma**	23
**Myelolipoma/lipoma**	31
**Hematoma**	5
**Angioma**	3
**Schwannoma**	4
**Lymphoma**	3
**Other types**	10

Supplementary Table 2: Classification results of external validation dataset 1: Metastasis

Lesion range	Method	Total adrenal glands	Prevalence	Accuracy	Sensitivity	Specificity	PPV	NPV	P-value
**All**	Model	89	0.607	0.978(0.947 - 1.000)	0.981(0.946 - 1.000)	0.971(0.916 - 1.000)	0.981(0.946 - 1.000)	0.971(0.916 - 1.000)	1.000
	Report			0.978(0.947 - 1.000)	0.981(0.946 - 1.000)	0.971(0.916 - 1.000)	0.981(0.946 - 1.000)	0.971(0.916 - 1.000)	
**10-15 mm**	Model	3	0.667	1.000(1.000 - 1.000)	1.000(1.000 - 1.000)	1.000(1.000 - 1.000)	1.000(1.000 - 1.000)	1.000(1.000 - 1.000)	1.000
	Report			1.000(1.000 - 1.000)	1.000(1.000 - 1.000)	1.000(1.000 - 1.000)	1.000(1.000 - 1.000)	1.000(1.000 - 1.000)	
**>15 mm**	Model	86	0.605	0.977(0.945 - 1.000)	0.981(0.943 - 1.000)	0.971(0.914 - 1.000)	0.981(0.943 - 1.000)	0.971(0.914 - 1.000)	1.000
	Report			0.977(0.945 - 1.000)	0.981(0.943 - 1.000)	0.971(0.914 - 1.000)	0.981(0.943 - 1.000)	0.971(0.914 - 1.000)	

Supplementary Table 3: Classification results of external validation dataset 1: Adenoma

Lesion range	Method	Total adrenal glands	Prevalence	Accuracy	Sensitivity	Specificity	PPV	NPV	P-value
**All**	Model	1211	0.534	0.981(0.973 - 0.989)	0.997(0.993 - 1.000)	0.963(0.947 - 0.978)	0.968(0.955 - 0.982)	0.996(0.991 - 1.000)	1.000
	Report			0.985(0.978 - 0.992)	1.000(1.000 - 1.000)	0.968(0.954 - 0.983)	0.973(0.961 - 0.985)	1.000(1.000 - 1.000)	
**5-10 mm**	Model	2	0.5	0.000(0.000 - 0.000)	0.000(0.000 - 0.000)	0.000(0.000 - 0.000)	0.000(0.000 - 0.000)	0.000(0.000 - 0.000)	1.000
	Report			1.000(1.000 - 1.000)	1.000(1.000 - 1.000)	1.000(1.000 - 1.000)	1.000(1.000 - 1.000)	1.000(1.000 - 1.000)	
**10-15 mm**	Model	76	0.513	0.961(0.917 - 1.000)	0.974(0.925 - 1.000)	0.946(0.873 - 1.000)	0.950(0.882 - 1.000)	0.972(0.919 - 1.000)	1.000
	Report			1.000(1.000 - 1.000)	1.000(1.000 - 1.000)	1.000(1.000 - 1.000)	1.000(1.000 - 1.000)	1.000(1.000 - 1.000)	
**>15 mm**	Model	1133	0.536	0.984(0.977 - 0.991)	1.000(1.000 - 1.000)	0.966(0.950 - 0.981)	0.971(0.958 - 0.984)	1.000(1.000 - 1.000)	1.000
	Report			0.984(0.977 - 0.991)	1.000(1.000 - 1.000)	0.966(0.950 - 0.981)	0.971(0.958 - 0.984)	1.000(1.000 - 1.000)	

Supplementary Table 4: Classification results of external validation dataset 1: Other pathological types

Lesion range	Method	Total adrenal glands	Prevalence	Accuracy	Sensitivity	Specificity	PPV	NPV	P-value
**All**	Model	503	0.531	0.990(0.981 - 0.999)	1.000(1.000 - 1.000)	0.979(0.960 - 0.997)	0.982(0.966 - 0.998)	1.000(1.000 - 1.000)	1.000
	Report			0.986(0.976 - 0.996)	0.996(0.989 - 1.000)	0.975(0.954 - 0.995)	0.978(0.960 - 0.995)	0.996(0.987 - 1.000)	
**10-15 mm**	Model	2	0.5	1.000(1.000 - 1.000)	1.000(1.000 - 1.000)	1.000(1.000 - 1.000)	1.000(1.000 - 1.000)	1.000(1.000 - 1.000)	1.000
	Report			1.000(1.000 - 1.000)	1.000(1.000 - 1.000)	1.000(1.000 - 1.000)	1.000(1.000 - 1.000)	1.000(1.000 - 1.000)	
**>15 mm**	Model	501	0.531	0.990(0.981 - 0.999)	1.000(1.000 - 1.000)	0.979(0.960 - 0.997)	0.982(0.966 - 0.998)	1.000(1.000 - 1.000)	1.000
	Report			0.986(0.976 - 0.996)	0.996(0.989 - 1.000)	0.974(0.954 - 0.995)	0.978(0.960 - 0.995)	0.996(0.987 - 1.000)	
